# Piloerection persists throughout repeated exposure to emotional stimuli

**DOI:** 10.1371/journal.pone.0309347

**Published:** 2024-09-18

**Authors:** Jonathon McPhetres, Hui H. Gao, Nicole Kemp, Bhakti Khati

**Affiliations:** Department of Psychology, Durham University, Durham, United Kingdom; Northwestern University Feinberg School of Medicine, UNITED STATES OF AMERICA

## Abstract

It is often suggested that piloerection, or goosebumps, is primarily triggered by emotional experience—theoretical perspectives place a heavy emphasis on experiencing novelty and surprise. However, the two studies described here challenge this perspective, demonstrating that the incidence of piloerection is not contingent upon exposure to novel stimuli and is disconnected from self-reported emotions. Study 1 (N = 80) shows that piloerection was not more likely to occur among individuals exposed to unfamiliar stimuli compared to those with prior exposure. Additionally, self-reported emotions were not correlated with observed piloerection. Study 2 (N = 27) found that piloerection persists throughout multiple exposures to identical stimuli. Importantly, the trajectories of observed piloerection and self-reported emotions diverged greatly. These findings challenge the common view that piloerection—unlike self-reported goosebumps and chills—is driven by emotional experience, suggesting that it may not be as closely connected to emotional experiences as previously theorised.

## Introduction

Piloerection, commonly referred to as goosebumps, arises from the contraction of tiny muscles at the base of hair follicles, leading to the erection of hairs and the formation of bumps on the skin. In the animal kingdom, this response serves well-defined roles in thermoregulation and social signalling. However, the study of piloerection in humans has received comparatively little attention. This oversight is not due to a lack of interest but because it is often overshadowed by the study of subjective phenomena such as ’the chills,’ frequently associated with emotional reactions to music [1]. Even though piloerection is often not directly observed in these studies, the chills are commonly defined as including piloerection [2, 3]. Thus, there’s a tendency to extrapolate the chills-emotions associations to piloerection, and we are left with a dearth of research on the physiological phenomenon itself. Against this backdrop, our research is designed to investigate piloerection directly, allowing us to test multiple theoretical assumptions that have long gone untested.

First, it is a common assumption that piloerection serves as a physical marker of emotional experiences, notably those triggered by novel or surprising events. This concept is ingrained in various theories, such as Keltner & Haidt’s [4] notion of "epistemic emotions." Epistemic emotions—surprise, curiosity, awe—are described as those arise when *new information* disrupts our established cognitive schemas. Such emotions have been anecdotally linked to goosebumps across a range of studies [5, 6]. Similarly, the Knowledge Instinct theory [7] theorizes that encountering *novel* entities or information in our environment evokes an emotional response, leading to chills. The theory of contrastive valence [8] also posits that *unexpected changes* in music, volume, or tone trigger an instinctual fight-or-flight reaction because, in the wild, such changes can signal impending danger. This overlap between our experience of surprising changes and the sympathetic nervous system is theorised to elicit chills.

Therefore, because the chills are often defined as including piloerection, the assumption in the wider literature—whether implicit or explicit—is that piloerection also results from the experience of surprise or novelty. According to this view, piloerection should be much more likely to occur in response to unfamiliar stimuli, or in response to stimuli with surprising and unexpected qualities. Further, because piloerection and these emotions are linked under this perspective, they should exhibit similar trajectories across repeated exposure to stimuli. For example, when surprise diminishes, piloerection should not occur.

On the other hand, a careful consideration of the literature reveals that the empirical evidence for a link between piloerection and emotions is actually quite limited. For example, a systematic review conducted by McPhetres & Zickfeld [9] highlights the lack of consistent correlations between piloerection and emotional responses, thereby casting doubt on the emotion-piloerection link more generally. Further, it may come as a surprise to learn that even widely cited claims purporting to demonstrate the link between piloerection and emotions have not been empirically substantiated. Take, for instance, the assertion that “in humans, piloerection shifted in its use, coming to occur regularly when we ourselves feel expanded beyond the boundaries of our skin…” (Keltner, [10], pages 446–447). Not only is the first part of this claim not even test*able*, because it refers to a point in our evolutionary history, but it is difficult to operationalise what it means to feel “expanded beyond the boundaries of our skin.” Another example is the claim that "piloerection may be a valid indicator of awe" [11]. However, upon critical inspection of the report, the actual correlation between piloerection and awe is never actually tested nor reported. Likewise, other studies claiming to show that “goosebumps” are related to emotions like awe have not objectively measured piloerection but, instead, rely on retrospective self-reports [5, 12]. Notably, the only study to directly investigate the connection between awe and piloerection discovered no correlation [13].

Thus, an alternative hypothesis is that piloerection may not be inherently connected to any specific emotional experiences at all; rather, it may occur in reaction to a broad range of environmental stimuli, independent of a person’s subjective appraisals of those stimuli. One way to test this hypothesis is to investigate whether piloerection would continue to persist through repeated exposures of certain stimuli, even though emotional reactions such as surprise diminish over time.

Our research comprises two studies that explore these possibilities. Study 1 compares piloerection responses among individuals with varying familiarity to a set of stimuli and assesses whether those stimuli rated as surprising or with "twist endings” are more likely to provoke piloerection. This allows us to test the implicit assumption that piloerection, like the chills, results from experiences involving surprise and novelty. Study 2 extends this inquiry by repeatedly exposing participants to the same stimuli, thereby mitigating the confounding influences of novelty and surprise, and examines whether piloerection and self-reported emotions track together or diverge over time.

## Study 1

### Method

#### Ethics and data collection

Written consent was obtained from participants. This study (and Study 2) was approved by the Durham University Psychology Ethics committee PSYCH-2021-12-06T16_03_28-mqbg73. Data were collected From 8 November 2021 through 26 August 2022.

#### Participants

Eighty participants were recruited from the psychology participant pool as well as from the surrounding community. There were 65 females and 15 males ranging in age from 18 to 50 (M = 21.00, SD = 5.38). Participants were compensated with either course credit, a prize draw, or cash. A power analysis indicates that a sample size of 80 with 7 within-person repetitions will provide 80% power to detect effects of *r*^2^ = .09 or greater.

#### Procedure

Participants arrived in a laboratory where they were connected to physiological equipment (Biopac MP160) and seated at a computer running e-Prime (v 2.0) in a private cubicle. In total, the experimental session took about 2 hours.

Following a baseline period, participants watched 7 videos intended to induce piloerection; the videos lasted about 25 minutes in total. After each video, participants answered a series of self-report questions. These questions included multiple emotion ratings rated on a scale from 1 (not at all) to 5 (extremely): emotional, exciting, frightening, sad scary, surprising, thrilling, and touching. Additionally, participants rated *unfamiliarity*: “Have you seen this video before?” coded as *Yes* (0) or *No* (1), and *surprise ending*: “Would you say the video has a surprise or twist ending?” recoded as *No* (0) or *Yes* (1). See supplementary materials for additional details about experimental design and missing data.

A series of cameras synced with Acqknowledge (v 5.0) objectively verified the presence of piloerection (see details in the supplementary materials). One camera was placed on the upper dorsolateral arm, one on the dominant dorsal calf, and one each on the dominant and non-dominant anterolateral thigh. Videos were viewed by coders in BORIS (v. 8), and the beginning of each piloerection episode was recorded at the nearest second. Two independent coders viewed each video and reached 100% agreement, and the first author then reviewed a random subset of the coding for quality checks.

### Results

In this study, 50% of participants experienced piloerection. Further details of the ratings for each video are presented in the supplementary materials. To examine the effects of prior exposure and the surprising nature of stimuli on piloerection, we carried out a series of mixed-effects regression models predicting piloerection likelihood (0 = no piloerection, 1 = piloerection) with the interaction between video (dummy codes) and either familiarity or twist endings. A model assessing the effect of video familiarity on piloerection explained minimal variance (see Table 1; *R*^2^ = .05). Notably, not having prior exposure *decreased* the likelihood of piloerection when watching ’Avengers: Endgame’ and ’Sandy Hook Promise,’ but this was not a general trend; the effect sizes were negligible for the other videos (less than 1% variance). Similarly, videos with unexpected or "twist" endings did not consistently increase piloerection likelihood (*R*^2^ = .04). Twist endings were associated with a *decreased* likelihood of piloerection only for ’Sandy Hook Promise’ and ’Thank You, Mom’.

**Table 1:** Likelihood of experiencing piloerection according to prior exposure and whether video had a twist ending.

	Likelihood of piloerection if			Likelihood of piloerection if video		
	video was previously unseen			has a twist ending		
	(R^2^ = .05)			(R^2^ = .04)		
Video	Odds Ratio	*p*	sr^2^	Log-odds	*p*	sr^2^
Avengers: Endgame	.265	.046	.01	.927	.932	< .01
Dear Brother	.498	.137	< .01	.502	.118	< .01
Thank you, Mom	1.738	.212	< .01	5.137	.034	< .01
Ripple	1.241	.635	< .01	2.070	.069	< .01
Sandy Hook Promise	.239	.008	.02	.353	.019	0.01
Shallow	1.661	.357	< 01	.257	.374	< .01
10-year-old singer	1.751	.204	< .01	2.259	.127	< .01

*Note*: Familiarity and *non-*twist endings were the reference conditions

sr^2^ indicates semi-partial r^2^.

Next, we examined whether self-reported emotions correlated with piloerection. In a mixed-effects logistic model, we entered each emotion rating simultaneously as predictors of piloerection likelihood with random intercepts for subject and video. As shown in Table 2, below, the model only explained about 7% of variance in piloerection likelihood. Only two emotion ratings correlated with piloerection: high ratings on “emotional” increased the likelihood of piloerection by about 93%, and higher ratings of “scary” decreased piloerection likelihood by about 33%.

**Table 2:** The correlation between self-reported emotion ratings and the likelihood of experiencing piloerection.

Effect	Odds Ratio	*p*	sr^2^
Emotional	1.839	.022	< .01
Exciting	1.243	.320	< .01
Frightening	1.814	.219	< .01
Sad	1.229	.460	< .01
Scary	.339	.038	< .01
Surprising	1.168	.406	< .01
Thrilling	1.328	.229	< .01
Touching	.912	.669	< .01

## Discussion

Our findings suggest that novelty and the element of surprise do not play substantial roles in inducing piloerection. Despite varying degrees of prior exposure and the presence of twist endings, the models accounted for a minimal portion of the variance in piloerection instances. Even commonly viewed videos like ’Avengers’ or ‘Shallow’ which had been seen by about half of participants, elicited piloerection in a significant portion of those with prior exposure, effectively countering the assumption that novelty or surprise are critical factors in piloerection responses. Second, there were no clear links between emotion ratings and the likelihood of experiencing piloerection. Specifically, only two out of eight emotion ratings were associated with the experience of piloerection, and these effect sizes were very small. If emotions are highly important, we would expect to see these emotions being very strongly associated with the experience of piloerection.

Of course, there are limitations to this study, including the fact that many of the videos were unfamiliar to participants. Further, among those who indicated prior exposure to a video, the level of familiarity may vary depending on how recently they had seen the content and how many times they had seen it. Study 2 aims to address these complexities observed in Study 1 by repeating exposure to the same stimuli and examining the persistence of piloerection, as well as its covariance with self-reported emotions.

## Study 2

### Method

#### Participants

We recruited 30 participants; 24 females and 5 males ranging in age from 18 to 50 (M = 19.93, SD = 0.88); demographics data for one participant was not recorded. Video recording data for 3 participants was lost due to a computer error, precluding piloerection analysis for those participants and reducing our total sample size to 27. A power analysis indicates that a sample size of 27 with 5 within-person repetitions will provide 80% power to detect effects of *r*^2^ = .23 or greater. Participants were compensated with either course credit or cash. Data were collected from 11 November 2022 through 3 March 2023.

#### Method

All setup, equipment, and analysis procedures were the same as in Study 1. However, the experimental procedure differed slightly because this study was designed to examine the effects of repeated exposure. First, following the baseline period, participants were randomly assigned to watch one of two videos from Study 1: “Thank you, Mom” (n = 16) and “10-year-old Singer” (n = 14) videos. These two videos were chosen because they were the among the most effective at eliciting piloerection during Study 1, because they were about the same length, and so that we had a variety of content. The video was then displayed five times in a row with a brief pause between exposures.

Second, participants rated several self-report questions after the first and final presentation, on a scale from 1 (not at all) to 5 (extremely): surprised, emotional, touched, excited, sad, chill, entertained, bored, annoyed, frustrated, intense, and engaged. Additionally, participants were asked whether they had previously seen the video (Yes or No). All participants were naïve except one, so this variable was not used in any analyses. Therefore, there was only a short pause after the second, third, and fourth presentations. In total, the experimental session took about 2 hours.

### Results

In total, 63% (N = 17) experienced piloerection, echoing the effectiveness of each video in Study 1. The videos did not differ in their ability to cause piloerection events (B = -.94, *p* = .690, *sr*^2^ = .017), so further analyses were collapsed across both videos.

**Fig 1:**
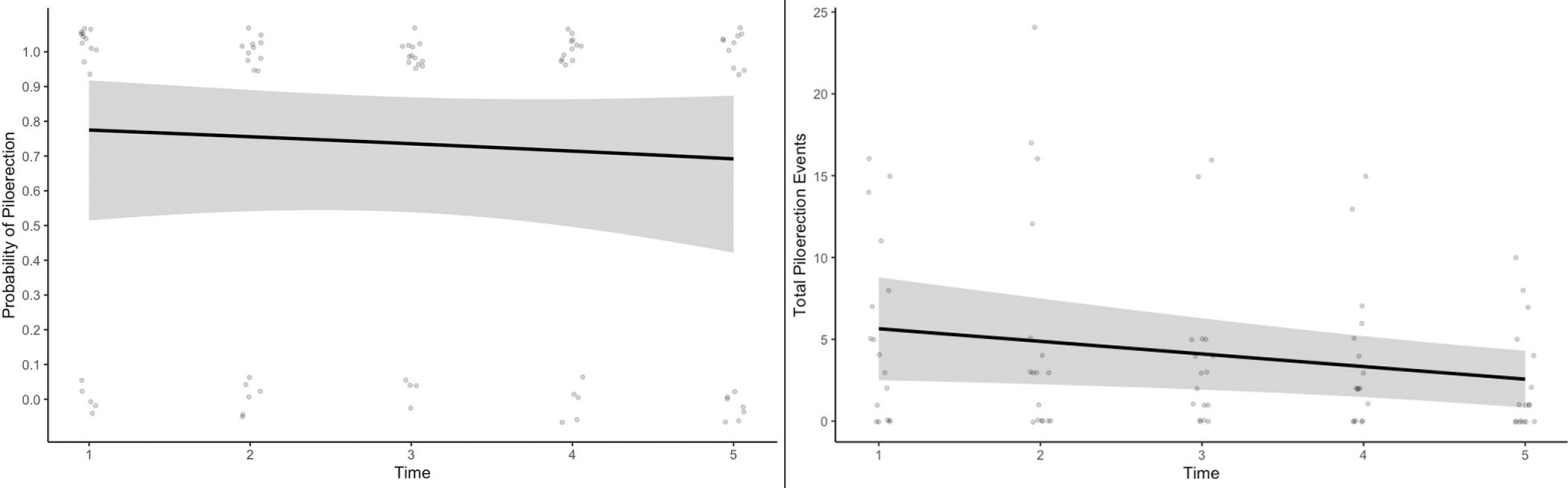
The likelihood of experiencing piloerection and the number of piloerection events were highly consistent over five exposures. *Note*: Shaded band indicates 95% CI.

First, piloerection events were still clearly present in most participants after five exposures (see Fig 1). A logistic mixed-effects regression model indicated no change in the likelihood of experiencing piloerection over repeated presentations, (Odds = .89, *p* = .57, *sr*^2^ = < .01). Of the 17 participants who experienced piloerection *at any point*, ten of them (59%) continued to experience piloerection during the fifth exposure.

While the number of piloerection events experienced by each person did decrease very slightly, this change is negligible (Fig 1, right pane). On average, 1.10 fewer piloerection events were experienced with each presentation (B = -1.10, *p* = .030, *sr*^2^ = .11), an extremely small effect size. Again, piloerection was still observed in most participants after five exposures. Further, very high correlations (see Table 3) were observed between the number of piloerection events during each presentation (average correlation of *r* = .72). Put differently, if a person experienced piloerection during one video, they continued to experience it during subsequent exposures.

**Table 3:** The correlation between number of piloerection episodes during each presentation of stimuli in Study 2.

Time	1	2	3	4	M	SD
**1**	-				5.35	5.61
**2**	.88***	-			5.35	7.31
**3**	.79***	.85***	-		3.82	4.77
**4**	.58*	.61**	.70**	-	3.65	4.47
**5**	.57*	.69**	.81***	.73***	2.35	3.24

***p < .001

**p < .01

*p < .05

**Fig 2:**
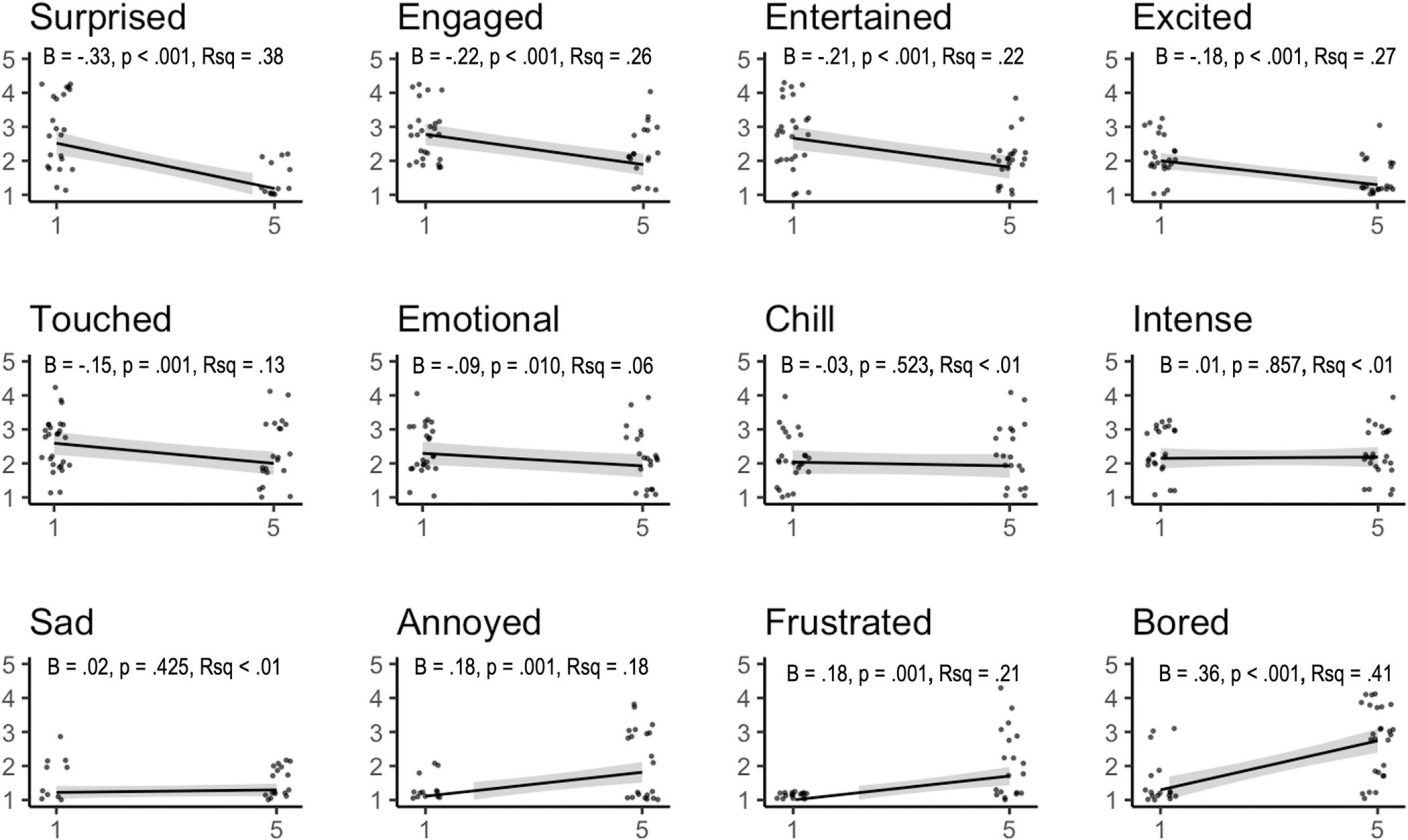
Change in self-report emotions over repeated presentation of stimuli in Study 2. *Note*: B indicates unstandardised coefficient; Rsq indicates model variance; shaded bands indicate 95% CI; points indicate raw data.

On the other hand, self-reported emotions changed markedly from the first to the final exposure (see Fig 2). With the exception of three items, each self-reported emotion showed large changes from the first to the final display: surprised, emotional, touched, excited, entertained, and engaged all *decreased* over time, while bored, annoyed, and frustrated *increased*. This is consistent with the idea that self-reported emotional and physiological changes are independent from one another.

However, because there was a slight decrease in the number of piloerection events (but not in the likelihood of experiencing piloerection) over the five exposures, we carried out an additional analysis to examine whether that change in an emotion (e.g., *surprise*) might predict a lower likelihood of experiencing piloerection during the fifth exposure. That is, to the extent that emotions diminish over multiple exposures, that digression might predict a decreased likelihood of piloerection after five exposures. To test this, we carried out a series of residual change models in which we predicted the likelihood of experiencing piloerection at Time 5 with the likelihood of experiencing piloerection at Time 1 and the *change* in emotion rating (calculated as Time 5 minus Time 1).

The full set of models is reported in Table 4, below. In short, none of the models explained significant amounts of variance in the likelihood of piloerection. Only two emotions had large effect sizes: to the extent that participants felt more *emotional* and more *sad*, their likelihood of experiencing piloerection was 3.46 and 6.42 times higher than baseline, respectively. Interestingly, a change in the emotion *surprised* only changed the likelihood of piloerection by about .68 times. Put differently, even when a participant’s experience of *surprise* decreased markedly, that participant’s likelihood of experiencing piloerection did not change substantially from Time 1 to Time 5.

**Table 4:** Residual change models predicting the likelihood of experiencing piloerection at Time 5 with the change in emotions from Time 1 to Time 5 controlling for piloerection at Time 1.

Term	Odds ratio	SE	*z*	*p*	Term	Odds ratio	SE	*z*	*p*
ΔSurprised	1.68	.41	1.28	.202	ΔEntertained	1.63	.49	.99	.324
Piloerection T1	4.35	.95	1.56	.120	Piloerection T1	3.48	.88	1.41	.158
ΔEmotional	4.46	.82	1.83	.067	ΔAnnoyed	0.52	.51	-1.30	.193
Piloerection T1	2.18	.90	.86	.388	Piloerection T1	3.20	.87	1.34	.181
ΔTouched	2.22	.60	1.34	.180	ΔBored	0.67	.37	-1.09	.277
Piloerection T1	2.62	.86	1.13	.260	Piloerection T1	3.41	.87	1.40	.161
ΔExcited	1.31	.54	.50	.621	ΔFrustrated	0.48	.54	-1.37	.172
Piloerection T1	2.72	.83	1.21	.225	Piloerection T1	3.59	.89	1.43	.152
ΔSad	7.42	1.21	1.66	.097	ΔIntense	1.28	.41	.60	.551
Piloerection T1	2.51	.88	1.04	.297	Piloerection T1	2.74	.83	1.22	.223
ΔChill	0.94	.48	-.13	.898	ΔEngaged	2.63	.58	1.66	.097
Piloerection T1	2.68	.84	1.17	.241	Piloerection T1	4.48	.95	1.58	.114

## Discussion

Our findings provide a critical perspective on the emotional underpinnings of piloerection. These data do not support the commonly held assumption that piloerection signifies emotional reactions to stimuli. Instead, piloerection can occur irrespective of a participant’s familiarity with the stimuli, and irrespective of its surprising nature. Further, the persistence of piloerection across repeated exposures to the same stimuli—despite a decrease in emotional responses such as surprise—suggests that the linkage between piloerection and emotions may not be as robust as posited by theories like those of Keltner & Haidt, the Knowledge Instinct, or Contrastive Valence.

The study yields several theoretical insights. First, it underlines a clear discrepancy between self-reported experiences and the physiological phenomena of piloerection. While participants did experience emotions that evolved over repeated exposures, piloerection remained relatively constant. This aligns with mounting evidence demonstrating that emotional experiences are orthogonal to piloerection [9, 13]. However, this discrepancy also suggests that self-reported goosebumps and the chills might represent a different phenomenon than objectively observed piloerection. This also accords, in a way, with the findings reported by [14], suggesting different trajectories for chills and piloerection over two exposures to stimuli. However, our study replicates and extends the basic finding by demonstrating this trajectory over five exposures and comparing it to multiple self-reported emotions. Thus, these different sets of findings strongly suggest that chills and piloerection represent different experiences and phenomena and should be discussed separately.

So how can we reconcile the persistent reporting of correlations between emotions and chills (or self-reported goosebumps) with more recent findings that challenge this association? A plausible explanation for this discrepancy lies in the concept of shared method variance. This concept suggests that self-reported measures tend to correlate more robustly with other self-reported metrics—a phenomenon long recognized by scholars. Termed the "crud factor" by Meehl [15], it posits that everything correlates with everything, “especially in the soft areas of psychology” (p. 327). Dang et al [16] reinforced this view, observing stronger congruence between self-reported data than between subjective accounts and objective measurements of the same phenomena. Applied to piloerection, it suggests that while individuals’ self-reported feelings of "goosebumps" or chills might align with their self-reported emotions, such alignments shouldn’t be misinterpreted as evidence of a link between the physical manifestation of piloerection and emotional experiences.

### Limitations and future directions

There are also some aspects that could be interpreted as limitations in our study. For instance, the study used a limited number of repeated exposures to stimuli (five), which was chosen to observe the acute effects on piloerection and emotions. It remains an open question whether piloerection and emotional responses might dissipate or persist differently over a more extended period, providing interesting implications for studies on long-term habituation and emotional resilience. In Study 1, even people who had previously seen the Avengers or Shallow videos still showed piloerection. At the time of this writing, these two videos had been available for 5 and 6 years, respectively, and they may be quite familiar to some participants (and still completely unfamiliar to others). Yet, they still elicited piloerection in a large proportion of viewers. This observation suggests that either the video contains piloerection-eliciting qualities, or the ability to continue to experience piloerection is an individual difference. While Study 2 did not measure personality or individual differences, it supports the hypothesis that the video itself contains piloerection-inducing qualities (e.g., because participants were randomly assigned to the videos, piloerection persisted throughout multiple exposures, etc). However, future research is needed on the relation between personality and piloerection.

The sex composition of our sample was predominantly female, which might also be considered a limitation on generalizability. However, additional analyses indicate that removing male participants does not alter the results or interpretations (see S3 and S4 Tables). Nonetheless, there is no existing evidence or biological to suggest significant differences in piloerection reactivity between sexes. The predominantly female sample can be interpreted as more representative of the emotional experience of females, offering a focused perspective that could be expanded upon in future studies.

Additionally, self-reported emotions come with inherent issues such as demand characteristics or retrospective bias. For example, recalling emotions after a stimulus may not be as accurate as reporting emotions in real-time during a stimulus. Interestingly, however, if participants were responding in a manner they thought was expected (e.g., reporting more boredom over time), they did not seem to take cues from their piloerection responses. Participants were informed that piloerection was being monitored, so if demand characteristics were a concern, it would be equally plausible for participants to report in line with the piloerection they were experiencing (e.g., less boredom, more interest). This highlights an intriguing point: recent studies have found that individuals are largely unaware of their piloerection [17]. This could mean that participants in our study either did not notice their piloerection and reported their emotions honestly, which is more plausible, or they chose to ignore their piloerection and report their experiences differently despite it, which seems less plausible.

Despite these considerations, our study makes several specific contributions to our understanding of piloerection in humans. First, we demonstrate that piloerection persists through repeated exposure, an insight useful for future researchers when selecting stimuli. This persistence suggests that researchers can use the same stimuli repeatedly rather than seeking novel stimuli for each exposure or for each participant. Second, the separate trajectory of piloerection and emotions implies that these phenomena are not strongly connected. Third, our findings indicate that piloerection is not specifically linked to “knowledge emotions” such as surprise or novelty, contributing to recent research (e.g., McPhetres & Shtulman, 2021) that questions the relation between piloerection and emotions like awe.

### Conclusions

The discourse on piloerection in humans has often been dominated by an emotional narrative, a stark contrast to the emphasis on piloerection as an environmental response in animal studies. This emotion-centric view in humans could overshadow other potential triggers or functions of piloerection (see, for example [18], suggesting that an emotional lens might not fully capture the essence of this complex phenomenon. Indeed, numerous large-scale meta-analyses [19, 20] argue against a direct congruence between physiological reactions and emotional states. This prompts the question of why piloerection should have been considered an emotional indicator in the first place.

In sum, our grasp of piloerection in humans remains limited, overshadowed by a preoccupation with self-reported chills. Slowly, emerging research is casting new light on this physiological response, revealing nuances that extend beyond the realm of emotions. It is becoming increasingly apparent that piloerection may not be as intimately connected to psychological phenomena as once posited.

## Supporting information

S1 Table Summary statistics for each video in Study 1. (DOCX)

S2 Table Linear mixed-effects regression models results from Study 1 using unfamiliarity to predict number of piloerection episodes. (DOCX)

S3 Table Predicting probability and number of piloerection events of five exposures in the female-only sample and the combined male/female sample. (DOCX)

S4 Table Changes in emotions from exposures one to five for females only. (DOCX)
