# Supplementary material for: Piloerection persists throughout repeated exposure to emotional stimuli
Source: PLoS One. 2024 Sep 18;19(9):e0309347. doi: 10.1371/journal.pone.0309347 (PMC11410212; doi:10.1371/journal.pone.0309347)
Supplement: S1 Table — (DOCX) [file pone.0309347.s001.docx]

**S1 Table.** Summary statistics for each video in Study 1.

| **Video Title** | **Familiarity** | | **Surprise ending or twist?** | |
| --- | --- | --- | --- | --- |
|  | **Familiar** | **Unfamiliar** | **No** | **Yes** |
| **Avengers** | 42 | 34 | 65 | 11 |
| **Dear Brother** | 2 | 73 | 14 | 61 |
| **Thank you, Mom** | 3 | 72 | 58 | 17 |
| **Ripple** | 7 | 68 | 16 | 59 |
| **Sandy Hook Promise** | 11 | 65 | 7 | 69 |
| **Shallow** | 36 | 41 | 72 | 5 |
| **10-year-old singer** | 3 | 74 | 45 | 32 |
