# Supplementary material for: Piloerection persists throughout repeated exposure to emotional stimuli
Source: PLoS One. 2024 Sep 18;19(9):e0309347. doi: 10.1371/journal.pone.0309347 (PMC11410212; doi:10.1371/journal.pone.0309347)
Supplement: S2 Table — (DOCX) [file pone.0309347.s002.docx]

**S2 Table.** Linear mixed-effects regression models results from Study 1 using unfamiliarity to predict number of piloerection episodes.

|  | Total number of piloerection events if  video was previously unseen  (R^2^ = .03) | | |  | Total number of piloerection events if  video has a twist ending  (R^2^ = .02) | | |
| --- | --- | --- | --- | --- | --- | --- | --- |
| **Video** | **Log-odds** | **p** | **sr^2^** |  | **B** | **p** | **sr^2^** |
| Avengers: endgame | -0.816 | 0.268 | < .01 |  | 0.467 | 0.726 | < .01 |
| Dear Brother | -1.42 | 0.012 | .01 |  | -1.033 | 0.043 | < .01 |
| Thank you, Mom | -0.674 | 0.227 | < .01 |  | 0.137 | 0.886 | < .01 |
| Ripple | -0.059 | 0.918 | < .01 |  | 0.219 | 0.672 | < .01 |
| Sandy Hook Promise | -1.589 | 0.007 | .01 |  | -1.097 | 0.025 | < .01 |
| Shallow | 0.74 | 0.285 | < .01 |  | -0.081 | 0.962 | < .01 |
| 10-year-old singer | 0.025 | 0.964 | < .01 |  | 0.935 | 0.179 | < .01 |

*Note:* Familiarity and *non*-twist ending are the reference conditions; R^2^ indicates total model variance; sr^2^ indicates semipartial r-squared.
