# Supplementary material for: Piloerection persists throughout repeated exposure to emotional stimuli
Source: PLoS One. 2024 Sep 18;19(9):e0309347. doi: 10.1371/journal.pone.0309347 (PMC11410212; doi:10.1371/journal.pone.0309347)
Supplement: S3 Table — (DOCX) [file pone.0309347.s003.docx]

**S3 Table.** Predicting probability and number of piloerection events of five exposures in the female-only sample and the combined male/female sample.

| **Predicting piloerection probability over time** | | | | | | |
| --- | --- | --- | --- | --- | --- | --- |
|  | **Females Only** | |  | **Combined Sample** | |  |
|  | **OR** | **P** |  | **OR** | **P** |  |
| **Time** | 1.05 | .820 |  | .89 | .570 |  |
|  |  |  |  |  |  |  |
| **Predicting number of piloerection events over time** | | | | | | |
|  | **Females Only** | |  | **Combined Sample** | |  |
|  | **B** | **P** |  | **B** | **P** |  |
| **Time** | -.75 | .080 |  | -1.10 | .030 |  |
|  |  |  |  |  |  |  |
