# Supplementary material for: Piloerection persists throughout repeated exposure to emotional stimuli
Source: PLoS One. 2024 Sep 18;19(9):e0309347. doi: 10.1371/journal.pone.0309347 (PMC11410212; doi:10.1371/journal.pone.0309347)
Supplement: S4 Table — (DOCX) [file pone.0309347.s004.docx]

**S4 Table.** Changes in emotions from exposures one to five for females only.

| **Term** | **Est.** | **p** | **Rsq** | **upper.CL** | **lower.CL** |
| --- | --- | --- | --- | --- | --- |
| Surprised | -0.333 | 0 | 0.38112791 | 0.56708071 | 0.19582805 |
| Engaged | -0.222 | 0 | 0.2625712 | 0.46467369 | 0.08990218 |
| Entertained | -0.213 | 0 | 0.22318345 | 0.42728979 | 0.0613231 |
| Excited | -0.176 | 0 | 0.26566674 | 0.46752606 | 0.09230568 |
| Touched | -0.148 | 0.001 | 0.13120992 | 0.32925841 | 0.01231265 |
| Emotional | -0.093 | 0.01 | 0.06302679 | 0.23859794 | 0.00055991 |
| Chill | -0.028 | 0.523 | 0.0047083 | 0.11517898 | 2.5132E-05 |
| Intense | 0.009 | 0.857 | 0.00061336 | 0.09914096 | 2.0457E-05 |
| Sad | 0.019 | 0.425 | 0.00730258 | 0.12409081 | 2.8657E-05 |
| Annoyed | 0.176 | 0.001 | 0.17867588 | 0.38212543 | 0.03404789 |
| Frustrated | 0.176 | 0.001 | 0.21338608 | 0.41764255 | 0.05483018 |
| Bored | 0.361 | 0 | 0.40875702 | 0.58932144 | 0.22408927 |
